# National Trends in HIV Pre-Exposure Prophylaxis Dispensing to Young Adults, 2016–2023

**DOI:** 10.1007/s11606-025-09574-8

**Published:** 2025-05-09

**Authors:** Nina E. Hill, Sijia He, Kao-Ping Chua

**Affiliations:** 1 Susan B Meister Child Health Evaluation and Research Center, Department of Pediatrics, University of Michigan Medical School, Ann Arbor, MI USA; 2 Department of Internal Medicine, University of Michigan, Ann Arbor, MI USA; 3 National Clinician Scholars Program at IHPI, Ann Arbor, MI USA

**Keywords:** HIV prevention, Pre-exposure prophylaxis, Young adults

## Abstract

**Background:**

U.S. young adults account for a disproportionate share of new HIV diagnoses. Despite this, no national study has examined recent trends in HIV pre-exposure prophylaxis (PrEP) dispensing to this age group.

**Objective:**

To assess trends in PrEP dispensing to young adults from 2016 to 2023.

**Design:**

Cross sectional study of the IQVIA Longitudinal Prescription Database.

**Participants:**

Young adults aged 18–25 years.

**Main Measures:**

Quarterly PrEP dispensing rate (number of young adults with dispensed PrEP prescriptions per 100,000 U.S. young adults), quarterly PrEP initiation rate (number of young adults with a new dispensed PrEP prescription per 100,000 U.S. young adults), and quarterly mean PrEP persistence (number of days with an active PrEP prescription in the 180 days after the new PrEP prescription ended among those initiating therapy). Joinpoint regression was used to identify slope changes and calculate the quarterly percent change (QPC). Subgroup analyses were conducted by age group.

**Key Results:**

Analyses included 1,450,296 PrEP prescriptions dispensed between January 1, 2016-December 31, 2023 to 239,780 young adults. The quarterly PrEP dispensing rate increased sharply between 2016–2018, from 26.4 to 100.7 prescriptions per 100,000 people (QPC: 12.5%, 95% CI: 10.0 to 15.1). Afterwards, this rate increased gradually to 208.4 prescriptions per 100,000 people in 2023 (QPC: 3.5%, 95% CI: 2.7 to 4.3). Trends in the quarterly PrEP initiation rate were qualitatively similar. From 2016 to 2023, quarterly mean persistence decreased from 111.6 to 98.4 days (QPC: -0.4%, 95% CI: -0.6, -0.2). Compared with patients aged 22–25 years, those aged 18–21 years had lower quarterly PrEP dispensing and initiation rates throughout 2016–2023. Persistence was lower in these younger patients in 2016, but the gap narrowed over time.

**Conclusions:**

Dispensing and initiation of PrEP among young adults is increasing, but treatment episodes are shortening. Efforts to promote retention in therapy are needed.

**Supplementary Information:**

The online version contains supplementary material available at 10.1007/s11606-025-09574-8.

## Introduction

HIV pre-exposure prophylaxis (PrEP) is defined as a medication regimen designed to reduce the risk of HIV transmission. These regimens can involve daily or on-demand use of medications as well as periodic injections. When taken as prescribed, PrEP decreases the risk of HIV transmission from sex by 99%.^1^ PrEP is recommended for sexually active, HIV-negative adolescents and adults with risk factors for HIV transmission in the last six months including two or more sexual partners, inconsistent or no condom use, one or more sexual partners with HIV, bacterial sexually transmitted infection, or engagement in commercial sex work.^2^ Despite the efficacy and safety of PrEP, only an estimated 36% of eligible patients use PrEP.^3^ Prior literature suggests the rate of PrEP use is particularly low among younger patients, non-Hispanic Black and Latino patients, publicly insured patients, and patients residing in the Southern U.S.^3–5^

Young adults account for a disproportionate share of new HIV diagnoses in the U.S.^6–8^ This age group may be prone to PrEP underuse since they face numerous barriers to accessing health care, such as lack of experience navigating the health care system and high rates of uninsurance. Despite these considerations, recent national data on trends in PrEP dispensing to young adults are lacking. Prior national studies have described increasing PrEP uptake among individuals aged 13–19 years and those aged 18 or older, but these studies were not specific to young adults.^3,9,10^ Additionally, there are few studies on trends in PrEP initiation or the degree to which patients who initiate PrEP continue therapy (PrEP “persistence”) in any age group. Among the studies on these topics that do exist, none were specific to young adults or used all-payer national data.^11^

The primary objective of this study was to evaluate trends in PrEP dispensing, initiation, and persistence among U.S young adults from 2016–2023. The secondary objective was to evaluate heterogeneity in these trends among subgroups defined by age group and Census region. To achieve these objectives, we analyzed data from an all-payer national prescription dispensing database.

## Methods

### Data Sources

We utilized the IQVIA Longitudinal Prescription Database (“IQVIA prescription database”) to identify patients with dispensed prescriptions for HIV PrEP. This database captures more than 93% of prescriptions at retail pharmacies and 60–85% of prescriptions at mail-order pharmacies in the United States. Data elements include prescription information (medication type, method of payment), patient demographics (age, region), and prescriber specialty. The database lacks information on race, ethnicity, or prescription indication.

To identify patients with diagnosis codes for HIV or hepatitis B, the IQVIA Longitudinal Prescription Database was linked to the IQVIA Medical Claims Database. The latter includes unadjudicated claims submitted to insurers by providers and facilities across the US, and approximately 45% of Americans are captured at least once in a given year. Because data were deidentified, the University of Michigan Medical School Institutional Review Board exempted analyses from human subjects review. Informed consent was not required. This manuscript follows the STROBE guidelines for reporting cross-sectional studies.^12^

### Sample

We identified prescriptions for PrEP dispensed to US young adults aged 18 to 25 years from January 1, 2016, through December 31, 2023. Because the IQVIA Longitudinal Prescription Database does not include prescription indication, we used a validated algorithm to distinguish antiretroviral medications prescribed for presumed PrEP from those prescribed for HIV post-exposure prophylaxis (PEP) or treatment of HIV or hepatitis B.^13,14^ Under this algorithm, a PrEP prescription met 3 criteria: 1) the prescription was for either tenofovir disoproxil fumarate/emtricitabine (TDF/FTC) or tenofovir alafenamide/emtricitabine (TAF/FTC); 2) the prescription had at least a 29-day supply (as a shorter duration might imply that the intended use was PEP); and 3) patients had no diagnosis codes for HIV or hepatitis B and no dispensing of other HIV antiretrovirals prior to the fill date. We did not include prescriptions for cabotegravir, which was approved for PrEP in 2021, because they were rare. Only 1, 743, and 1,836 young adults had any dispensing of this drug in 2021, 2022, and 2023, respectively.

### Outcomes

We included three primary outcomes: 1) quarterly PrEP dispensing rate, 2) quarterly PrEP initiation rate, and 3) quarterly PrEP persistence. Quarterly PrEP dispensing rate was defined as the number of unique young adults with at least one dispensed PrEP prescription during the quarter per 100,000 U.S. individuals aged 18–25 years. The quarterly PrEP initiation rate was defined as the number of young adults with at least one new dispensed prescription for PrEP during the quarter among the same denominator, meaning there were no dispensed prescriptions for TDF/FTC or TAF/FTC in the 180 days before the index date. For both outcomes, we used U.S. Census Bureau data to obtain annual population denominators for young adults aged 18–25 years between 2016 and 2023 (see Appendix 1).

To calculate PrEP persistence, we first identified “PrEP initiation episodes”, which began on the date a new PrEP prescription was dispensed. For each initiation episode, we determined the active period of the new prescription (dispensing date through the dispensing date plus days supplied minus one). Persistence was defined as the number of days covered with an active PrEP prescription between two dates: the date after the active period of the new prescription ended, and this date plus 180 days. We chose to measure persistence as a continuous rather than binary variable, as the former provides more granular information and is easier to interpret.

For example, if the new prescription was dispensed on January 1, 2016, and was written for a 30-day supply, the new prescription was active between January 1, 2016, through January 30, 2016. If the patient had only 1 additional PrEP prescription dispensed between January 31, 2016, and July 28, 2016 (i.e., 180 days after January 30, 2016), and if this additional prescription were for a 30-day supply, then PrEP persistence would be 30 days for this patient. If multiple PrEP prescriptions were active on a given day, we only counted the day once when measuring PrEP persistence.

### Statistical Analyses

For each year during 2016–2023, we described characteristics of young adults with any PrEP dispensing as of the time of the earliest fill during the year. We also described prescription characteristics (medication name, method of payment, and prescriber specialty). We calculated the unadjusted percentage change in all characteristics between 2016 and 2023.

To assess trends in outcomes, we used joinpoint regression, a technique that empirically tests for slope changes in trends. We allowed for up to 3 joinpoints (i.e., models could have 0, 1, 2, or 3 joinpoints) and used permutation tests to select the model with the best fit. We divided the study period into 32 three month periods and calculated the quarterly percentage change (QPC) in each segment.

In subgroup analyses, we repeated analyses of PrEP prescriptions by age group (18–21 years, 22–25 years) and Census region (Northeast, Midwest, South, West). We obtained annual population denominators of young adults by age group and region from the Census Bureau **(**Appendix 1**)**. All analyses were performed using SAS version 9.4 (SAS Institute, Cary, NC) and Joinpoint software version 5.0.1 (National Cancer Institute).^15^ Two-sided hypothesis tests with α = 0.05 were used.

## Results

### Sample Characteristics

From January 2016 to December 2023, 1,450,296 PrEP prescriptions were dispensed to U.S. young adults aged 18–25 years. The 1,450,296 prescriptions were dispensed to 239,780 unique patients. At the time of each patients’ earliest fill during the study period, mean (SD) age was 22.4 (2.1) years, 160,797 (67.0%) of patients were aged 22–25 years, 209,370 (87.3%) were male, and 88,475 (36.9%) resided in the Southern U.S (Table 1).

**Table 1:** Demographic Characteristics of Young Adults with Dispensed Prescriptions for PrEP between 2016 and 2023

Characteristic	2016	2017	2018	2019	2020	2021	2022	2023	% change, 2016–2023
No. with ≥ 1 dispensed PrEP prescription	15,543	24,402	35,924	43,516	44,994	58,191	68,626	74,475	379.2
Mean (SD) age in years	22.9 (1.9)	22.8 (1.9)	22.7 (2.0)	22.6 (2.0)	22.6 (2.0)	22.6 (2.0)	22.6 (2.0)	22.6 (2.0)	−1.3
Age group									
Age 18–21, No. (%)	3,573 (23.0)	6,071 (24.9)	9,571 (8.9)	12,231 (28.1)	12,827 (28.5)	17,339 (29.8)	20,163 (29.4)	21,557 (29.0)	503.3
Age 22–25, No. (%)	11,970 (77.0))	18,331 (75.1)	26,353 (73.4)	31,285 (71.9)	32,167 (71.5)	40,852 (70.2)	48,463 (70.6)	52,918 (71.1)	342.1
Sex									
Female, No. (%)	1,441 (9.3)	2,117 (8.7)	3,199 (8.9)	4,146 (9.5)	4,524 (10.1)	5,972 (10.2)	7,141 (10.4)	8,297 (11.1)	475.8
Male, No. (%)	14,089 (90.7)	22,275 (91.3)	32,707(91.0)	39,344(90.4)	40,059(89.9)	52,208 (89.7)	61,466 (89.6)	66,143 (88.8)	369.5
Census region									
Northeast, No. (%)	4,676 (30.1)	7,300 (29.9)	9,872 (27.5)	11,113 (25.5)	10,138 (22.5)	12,606 (21.7)	15,033 (21.9)	16,160 (21.7)	245.6
Midwest, No. (%)	2,772 (17.8)	4,460 (18.3)	6,383 (17.8)	7,694 (17.7)	7,225 (16.1)	9,361 (16.1)	11,202 (16.3)	12,253 (16.5)	342.0
South, No. (%)	4,125 (26.5)	6,554 (26.9)	10,643 (29.6)	14,416 (33.1)	17,545 (39.0)	23,355 (40.1)	26,667 (38.9)	28,931 (38.9)	601.4
West, No. (%)	3,970 (25.5)	6,088 (24.9)	9,026 (25.1)	10,293 (23.7)	10,086 (22.4)	12,869 (22.1)	15,724 (22.9)	17,131 (23.0)	331.5

Of these 1,450,296 prescriptions, 1,028,521 (70.9%) were for TDF/FTC and 421,775 (29.1%) were for TAF/FTC. The most common methods of payment were private insurance (1,195,802; 82.5%) and Medicaid (231,135; 15.9%). The most common prescriber types were nurse practitioners (555,416, 38.6%) and general practice/family practice physicians (318,709, 22.1%), followed by internal medicine physicians (204,860, 14.2%) and physician assistants (153,698, 10.7%). Between 2016 and 2023, the number of PrEP prescriptions increased the most among emergency medicine physicians (1,054%), followed by nurse practitioners (661%) (Table 2).

**Table 2:** Characteristics of Dispensed PrEP Prescriptions for Young Adults between 2016 and 2023

Characteristic	2016	2017	2018	2019	2020	2021	2022	2023	% change, 2016–2023
No. dispensed PrEP prescriptions	58,673	97,041	141,659	173,836	182,727	229,242	271,731	295,387	403.4
Medication, No. (%)									
TAF/FTC	85 (0.1)	363 (0.4)	442 (0.3)	8,975 (5.2)	71,360 (39.1)	101,709 (44.4)	114,461 (42.1)	124,380 (42.1)	146,229.0
TDF/FTC	58,588 (99.9)	96,678 (99.6)	141,217 (99.7)	164,861 (94.8)	111,367 (61.0)	127,533 (55.6)	157,270 (57.9)	171,007 (57.9)	191.9
Payment method, No. (%)									
Private insurance	48,224 (82.2)	78,979 (81.4)	115,836 (81.8)	145,697 (83.8)	154,792 (84.7)	191,495 (83.5)	221,178 (81.4)	239,601 (81.1)	396.9
Medicaid	9,298 (15.9)	16,449 (17.0)	23,778 (16.8)	26,396 (15.2)	26,124 (14.3)	34,530 (15.1)	45,310 (16.7)	49,250 (16.7)	429.7
Medicare	458 (0.8)	692 (0.7)	825 (0.6)	1,089 (0.6)	1,090 (0.6)	1,754 (0.8)	2,863 (1.1)	3,698 (1.3)	707.4
Cash	675 (1.2)	909 (0.9)	1,217 (0.9)	647 (0.4)	716 (0.4)	1,427 (0.6)	2,338 (0.9)	2,838 (1.0)	320.4
Prescriber specialty, No (%)									
Internal medicine									
General Internal Medicine	11,778 (20.4)	18,230 (19.0)	24,138 (17.2)	26,327 (15.2)	23,514 (13.0)	30,169 (13.3)	35,380 (13.1)	35,324 (12.0)	199.9
Adult infectious disease	7,268 (12.6)	11,534 (12.0)	12,881 (9.2)	12,787 (7.4)	12,093 (6.7)	11,811 (5.2)	11,739 (4.3)	12,420 (4.2)	70.9
Pediatrics									
General pediatrics	1,476 (2.6)	2,491 (2.6)	3,932 (2.8)	5,343 (3.1)	4,757 (2.6)	5,769 (2.5)	6,941 (2.6)	7,167 (2.4)	385.6
Pediatric infectious disease	152 (0.3)	297 (0.3)	321 (0.2)	418 (0.2)	383 (0.2)	407 (2.5)	465 (0.2)	474 (0.2)	211.8
General practice	14,599 (25.2)	21,289 (22.2)	31,497 (22.4)	37,768 (21.8)	38,248 (21.2)	46,363 (20.4)	61,306 (22.7)	67,639 (23.0)	363.3
Emergency medicine	461 (0.8)	639 (0.7)	1,007 (0.7)	1,152 (0.7)	1,715 (1.0)	5,548 (2.4)	9,998 (3.7)	16,077 (5.5)	3,387.4
Nurse practitioner	15,847 (27.4)	29,397 (30.6)	48,906 (34.8)	66,381 (38.4)	75,255 (41.6)	93,517 (41.1)	108,622 (40.2)	117,491 (40.0)	641.4
Physician assistant	5,294 (9.2)	10,362 (10.8)	14,703 (10.5)	19,225 (11.1)	20,336 (11.2)	24,383 (10.7)	28,838 (10.7)	30,557 (10.4)	477.2
Other specialty	994 (1.7)	1,792 (1.9)	3,004 (2.1)	3,496 (2.0)	4,523 (2.5)	9,593 (4.2)	6,614 (2.5)	6,605 (2.3)	564.5
Unknown	14 (0.02)	53 (0.06)	73 (0.06)	80 (0.05)	29 (0.02)	5 (0.00)	18 (0.01)	11 (0.00)	−21.4

#### PrEP Dispensing Rate

**Figure 1:**
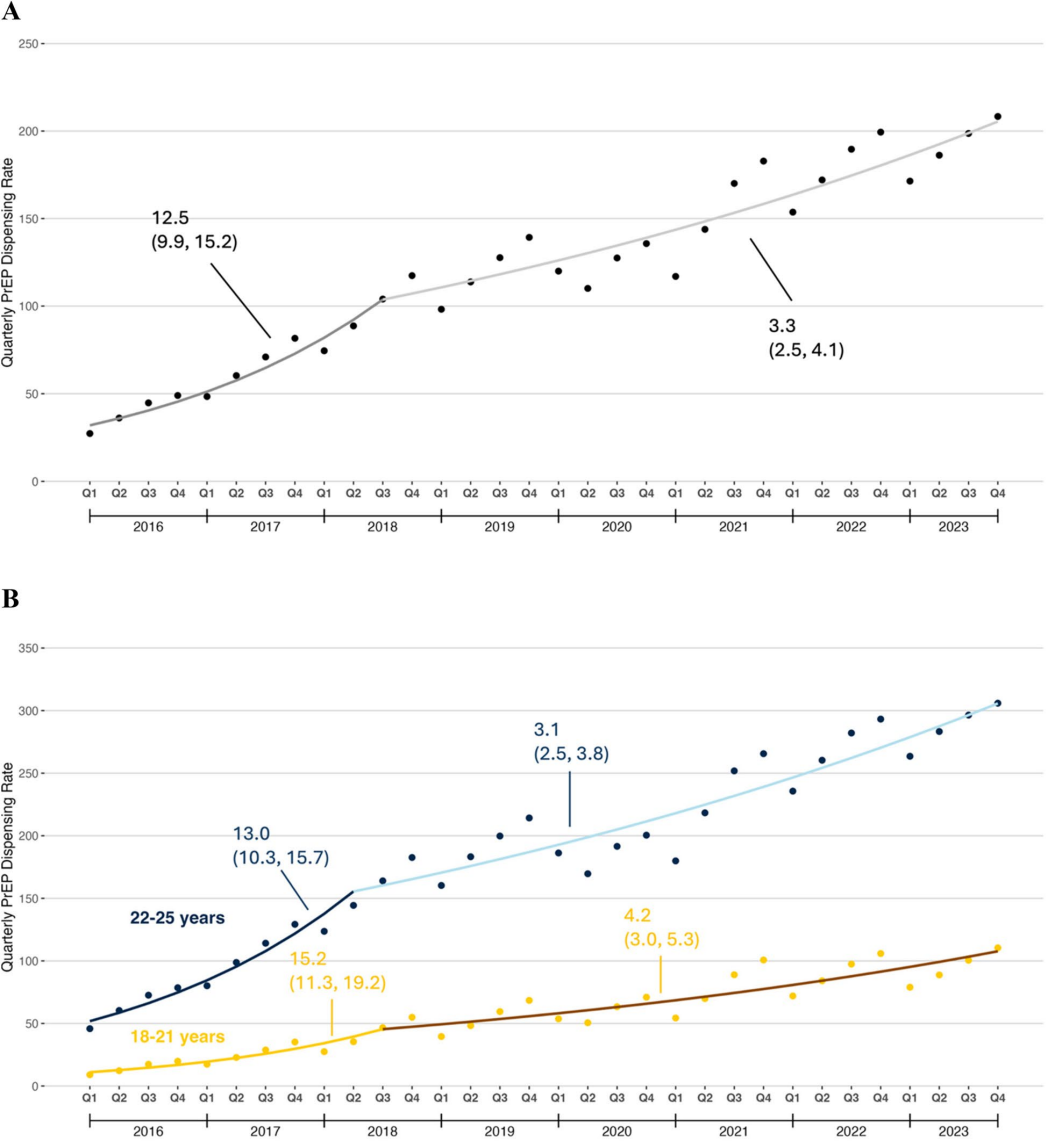

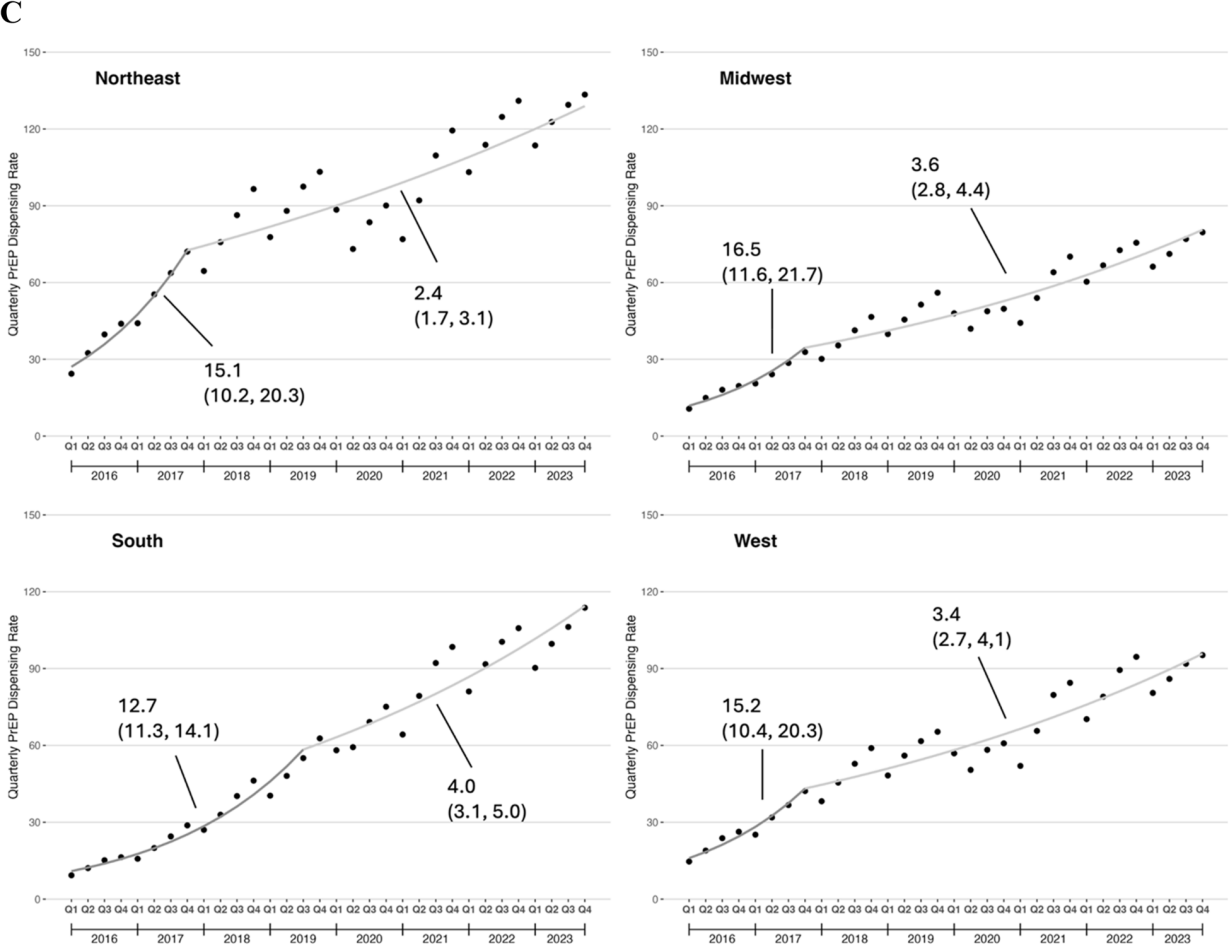
Quarterly PrEP dispensing rate among U.S. young adults aged 18–25 years, 2016–2023. **A** Overall trends; **B** Trends by age group; **C** Trends by U.S. Census region. The PrEP dispensing rate was defined as number of young adults with PrEP dispensing per 100,000 U.S. young adults on a quarterly basis.

Figure 1a displays trends in the PrEP dispensing rate among all young adults. From quarter 1 (Q1) 2016 through quarter 3 (Q3) 2018, this rate increased from 26.4 prescriptions per 100,000 people to 100.7 prescriptions per 100,000 people (QPC: 12.5%, 95% CI: 10.0 to 15.1). Afterwards, this rate increased more gradually to a peak of 208.4 prescriptions per 100,000 people in Q4 2023 (QPC: 3.5%, 95% CI: 2.7 to 4.3). There was a seasonal pattern to this rate, with the lowest rate in Quarter 1 and the highest in Quarter 4.

In Q1 2016, the PrEP dispensing rate was 9.0 prescriptions per 100,000 people among patients aged 18–21 years and 45.9 prescriptions per 100,000 people among patients aged 22–25 years. The PrEP dispensing rate increased through Q2 2018 for both age groups, though the absolute increase was greater in patients aged 22–25 than in patients aged 18–21. The gap in the quarterly PrEP dispensing rate widened substantially by Q4 2023 for patients aged 22–25 and patients aged 18–21 (Fig. 1b).

In Q1 2016, the PrEP dispensing rate was highest in the Northeast (24.4 prescriptions per 100,000 people) and lowest in the South (9.3 prescriptions per 100,000 people). By Q4 2023, the PrEP dispensing rate in the South was second only to the Northeast owing to rapid increases during the study period (Fig. 1c).

#### PrEP Initiation Rate

**Figure 2:**
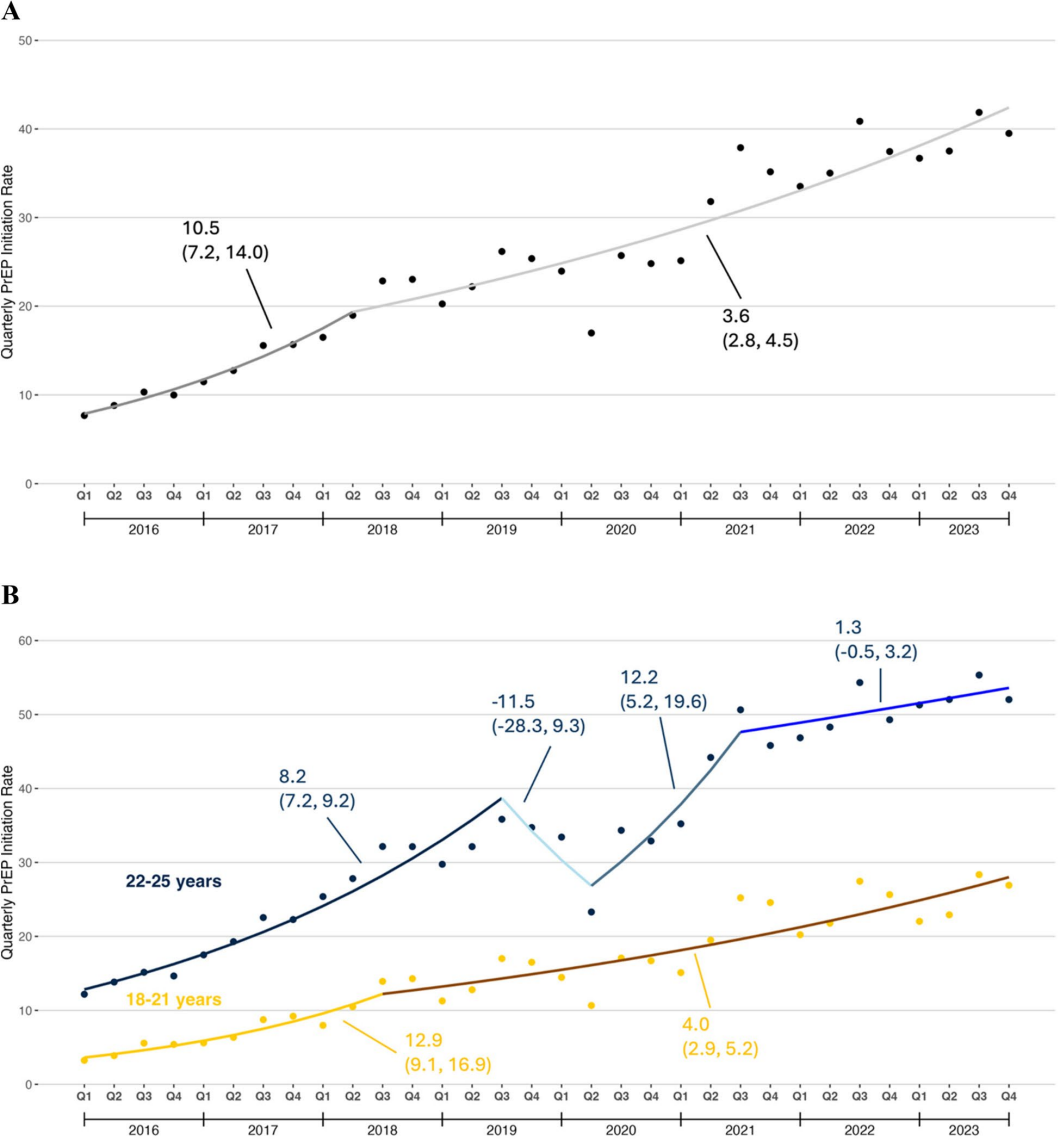

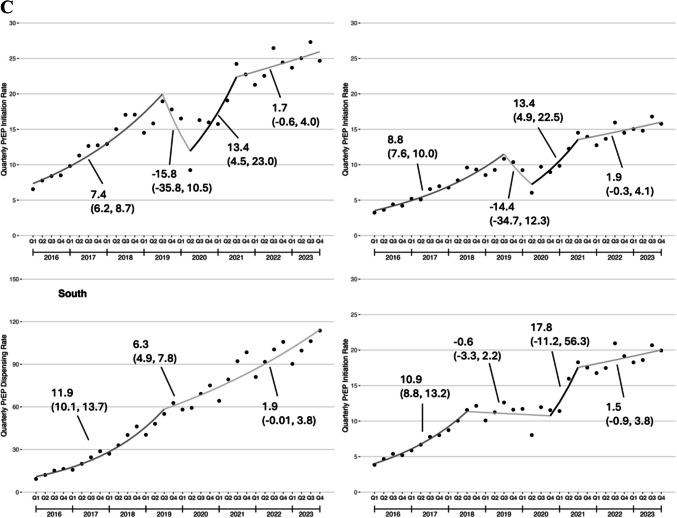
Quarterly PrEP initiation rate among U.S. young adults, 2016–2023. **A** Overall trends; **B** Trends by age group; **C** Trends by U.S. Census region. The PrEP initiation rate was defined as number of young adults with new PrEP prescriptions per 100,000 U.S. young adults on a quarterly basis. To be defined as a new PrEP prescription, the young adult had no PrEP dispensed in a 180 day lookback period.

Trend in the quarterly PrEP initiation rate were qualitatively similar to the quarterly PrEP dispensing rate, with rapid increases in PrEP initiation from 2016 through the middle of 2018, then more gradual increases afterwards (Fig. 2a). Similar to the PrEP dispensing rate, the PrEP initiation rate was lower among patients aged 18–21 years compared those with aged 22–25 years in Q1 2016, but this difference widened by Q4 2023 (Fig. 2b). Among patients in the South, PrEP initiation rates increased consistently throughout the study period. In contrast, there were declines during 2019–2020 in the Northeast and Midwest and a plateau between 2018 and 2020 in the West (Fig. 2c).

#### PrEP Persistence

**Figure 3:**
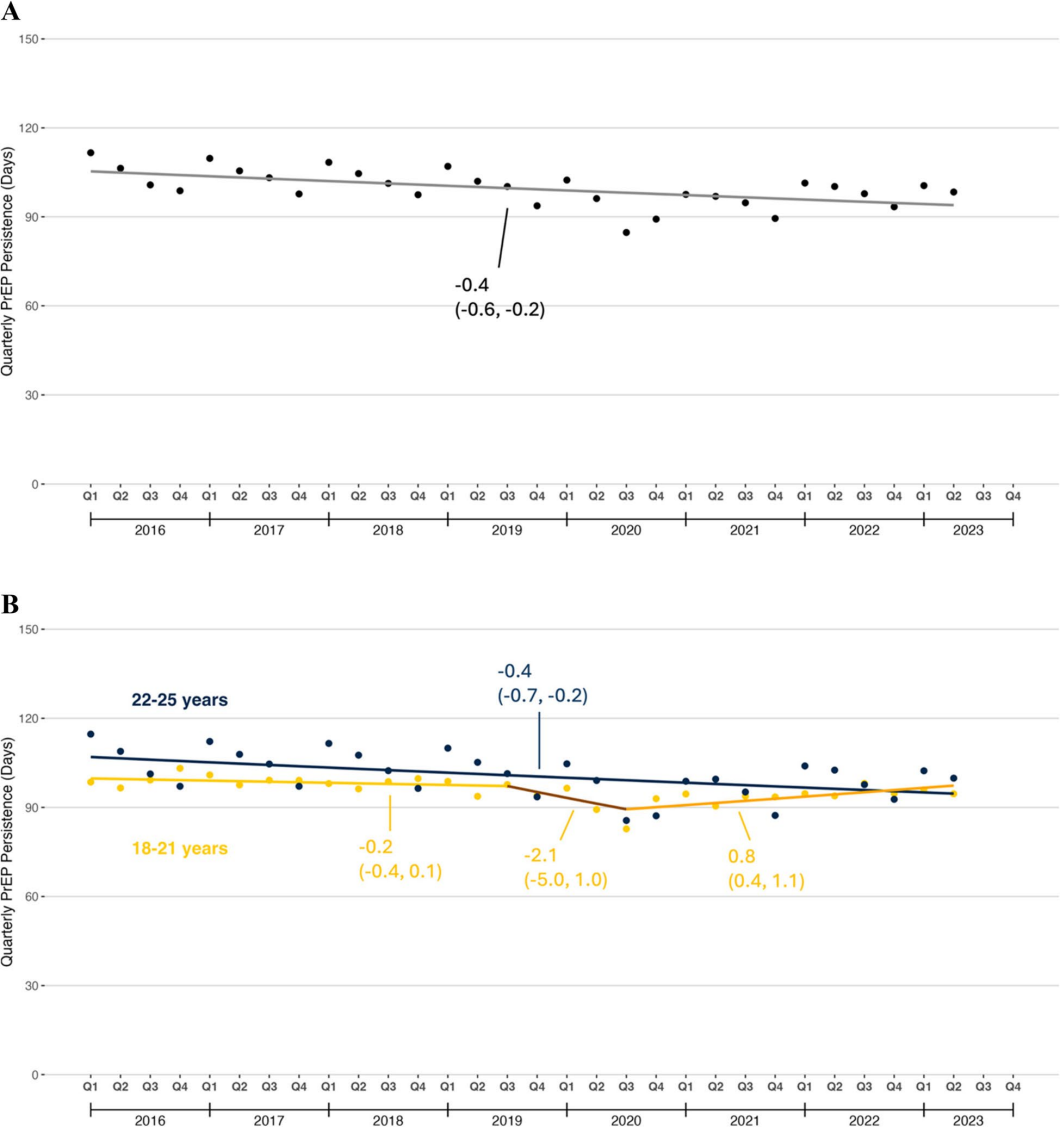

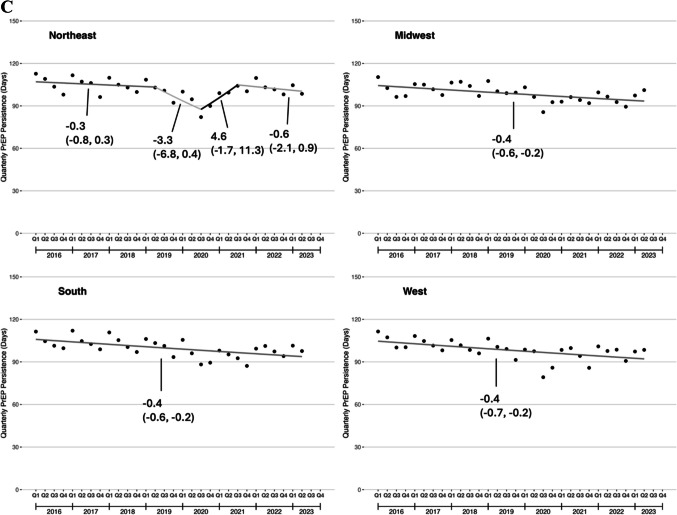
Quarterly Mean PrEP persistence among U.S. young adults, 2016–2023. **A** Overall trends; **B** Trends by age group; **C** Trends by U.S. Census region. PrEP persistence was defined as the number of days covered by an active PrEP prescription in the 180 days after the end of a new PrEP prescription.

Among young adults with new PrEP prescriptions, mean quarterly PrEP persistence gradually decreased from 111.6 to 98.4 days during the study period, with no joinpoint detected (QPC: −0.4%, 95% CI: −0.6, −0.2; Fig. 3a**)**. PrEP persistence was lower among patients aged 18–21 years compared to those aged 22–25 years at the beginning of the study period. The difference in PrEP persistence between patients aged 18–21 years and patients aged 22–25 years attenuated over time. Persistence steadily decreased during the study period among patients aged 22–25 years, whereas it increased starting in Q3 2020 for patients aged 18–21 years (Fig. 3b). There was little variation in the magnitude of PrEP persistence by Census region either at the beginning or end of the study period (Fig. 3c). In all 4 regions, mean quarterly PrEP persistence declined. No joinpoints were detected except in the Northeast, in which there was a decline from Q1 2019 to Q3 2020 and an increase from Q4 2020 to Q2 2021.

## Discussion

This analysis of a national all-payer database revealed that PrEP dispensing and initiation among young adults increased between 2016–2023. However, PrEP persistence decreased from a mean of 111.6 to 98.4 days.

To our knowledge, this is the first analysis to use comprehensive national prescription data to examine recent trends in both prevalent and incident use of PrEP among young adults, as well as changes in the duration of treatment episodes following PrEP initiation. Our findings are consistent with prior studies demonstrating increased PrEP dispensing to adolescents aged 13–19 years between 2018 and 2021 and increasing PrEP dispensing to adults of all ages between 2013 and 2023.^9,10^ More broadly, our findings of increasing PrEP dispensing are consistent with national data showing an increase in PrEP use among all U.S. individuals who would benefit from PrEP.

Our findings should be considered in light of the broader HIV epidemic in the U.S. From 2018 to 2022, new HIV infections among individuals aged 13–24 years decreased by 30%. This decrease may have been partly driven by the increase in PrEP dispensing to young adults. Importantly, the decline in new HIV infections does not necessarily indicate that the number of young adults who would benefit from PrEP is decreasing, as this number is affected not just by HIV prevalence but also by the prevalence of behaviors that increase risk of HIV transmission among HIV-negative young adults. Thus, our findings of increased PrEP dispensing should not be interpreted as definitive evidence that the gap between PrEP need and use is narrowing among young adults.

Though more young adults are initiating PrEP therapy, they are remaining in therapy for shorter periods of time. PrEP persistence decreased gradually over time and was not driven by the COVID-19 pandemic, as there was no joinpoint detected during 2020–2022. We offer three possible alternative explanations. First, declines in PrEP persistence may reflect changes in the characteristics of young adults who initiate PrEP. For example, it is possible that young adults initiating PrEP in more recent years are engaging in shorter periods of sexual activity and thus may not perceive the need for prolonged PrEP therapy. In potential support of this possibility, data suggest that fewer young adults report having multiple sexual partners.^16^ Second, barriers to follow-up after PrEP initiation may be increasing. Patients taking PrEP therapy require routine lab monitoring and clinical evaluation every 3 months.^17^ This follow-up often occurs through primary care, but primary care access has worsened over the past decade.^18,19^ Third, patients may be increasingly using PrEP on-demand rather than as a single daily dose.

Our subgroup analyses by age group demonstrated that the PrEP dispensing rate, initiation rate, and mean persistence was lower among patients aged 18–21 years compared to those aged 22–25 years in 2016. By the end of 2023, the gap in PrEP dispensing and initiation widened for patients aged 18–21 years compared to those 22–25 years, although differences in PrEP persistence narrowed. There are limited available data to explain this finding, since few reports compare healthcare utilization between patients aged 18–21 years versus 22–25 years. We offer two tentative explanations. First, patients aged 18–21 years are more likely to receive care from pediatric or adolescent health providers, who may be less comfortable with prescribing PrEP compared to adult providers.^20^ Second, patients aged 18–21 years are more likely to receive health insurance through dependent coverage from a parent compared to those aged 22–25 years.^21^ Young adults covered by their parents’ health insurance plans cite confidentiality concerns as a barrier to accessing sexual health services.^22,23^ Thus, privacy concerns may impede uptake of PrEP therapy to a greater degree among patients aged 18–21 years.

Subgroup analyses by region revealed that increases in the PrEP dispensing rate occurred rapidly in the South. This finding is encouraging given that the South has a disproportionate burden of HIV as well as the lowest PrEP-to-Need Ratio (the ratio of the number of PrEP users to the number of people newly diagnosed with HIV).^24,25^ The PrEP initiation rate in the South continuously increased during the COVID-19 pandemic, unlike the Northeast and Midwest, potentially because social distancing measures in the South were generally less widespread.^26^ Despite these findings, PrEP is still likely underused among young adults in the South. Public health efforts, such as increasing PrEP availability and social marketing campaigns,^27,28^ will be important to maintain and accelerate the progress in PrEP dispensing to young adults in the South. Policy efforts may also play a vital role in enhancing PrEP availability. For example, state legislation in Arkansas and Virginia allows pharmacists to prescribe PrEP. Such legislation might be extended to other Southern states.^29^

This analysis is subject to several limitations. First, due to data limitations, we were unable to stratify analyses by race, ethnicity, gender identity, or sexual orientation. Second, we chose not to stratify analyses by payer type. This analysis would require accurate data on the numbers of young adults with private insurance and Medicaid coverage during the study period. However, we are unaware of any data on these numbers during the COVID-19 pandemic, when insurance coverage of young adults shifted dramatically due to the combination of employer-based job loss and continuous Medicaid enrollment.^30^ Third, our definition of PrEP persistence assumed that patients took medications as prescribed, but some take these medications in a different manner (e.g., on-demand).^31^ Fourth, our estimates of the rate of PrEP dispensing and initiation used the underlying population of U.S. young adults as the denominator. These estimates likely underestimate the rate of PrEP dispensing and initiation among the subset of young adults who would benefit from PrEP, Fifth, because we did not have data on prescription indication, some prescriptions classified as PrEP may actually have been for antiretroviral therapy or HIV post-exposure prophylaxis. We mitigated this risk by using a previously described and validated algorithm for identifying PrEP prescriptions.^13^ Sixth, the IQVIA database does not capture PrEP prescriptions dispensed directly to patients during health care visits or prescriptions dispensed in closed pharmacies that only serve patients of specific hospitals or health care systems, such as Kaiser Permanente or the Veterans Administration. Thus, dispensing rates may be slightly underestimated.

## Conclusions

We demonstrate increasing PrEP dispensing and initiation rates for young adults in the U.S. between 2016 and 2023, but decreasing PrEP persistence. We also demonstrate lower rates of PrEP dispensing and initiation among young adults aged 18–21 years compared to 22–25 years. Future studies should investigate which interventions are most effective in promoting retention in PrEP therapy among young adults initiating PrEP and in promoting PrEP dispensing to patients aged 18–21 years.

## Supplementary Information

Below is the link to the electronic supplementary material.

Supplementary file1 (DOCX 47 KB)
