# Supplementary material for: National Trends in HIV Pre-Exposure Prophylaxis Dispensing to Young Adults, 2016–2023
Source: J Gen Intern Med. 2025 May 9;41(1):53–63. doi: 10.1007/s11606-025-09574-8 (PMC12855713; doi:10.1007/s11606-025-09574-8)
Supplement: Supplementary file 1 — Supplementary file1 (DOCX 47 KB) [file 11606_2025_9574_MOESM1_ESM.docx]

**Appendix 1:**

Table 1. Estimated population denominators for U.S. young adults aged 18-25 years

| **Denominator** | **2016** | **2017** | **2018** | **2019** | **2020** | **2021** | **2022** | **2023** |
| --- | --- | --- | --- | --- | --- | --- | --- | --- |
| **Overall** | 36,004,118 | 35,657,033 | 35,403,154 | 35,191,552 | 35,575,999 | 35,501,350 | 35,674,439 | 35,682,369 |
| **Age group** | | | | | | | | |
| 18-25 years | 34,877,363 | 34,541,140 | 34,295,206 | 34,090,226 | 34,473,452 | 34,425,948 | 34,639,989 | 34,883,367 |
| 18-21 years | 17,581,916 | 17,508,943 | 17,576,776 | 17,604,800 | 17,241,472 | 17,274,887 | 17,349,271 | 17,408,648 |
| 22-25 years | 17,307,482 | 17,052,507 | 16,755,331 | 16,533,676 | 17,231,980 | 17,151,061 | 17,290,718 | 17,474,719 |
| **Region** | | | | | | | | |
| Northeast | 12,225,154 | 12,064,239 | 11,923,878 | 11,737,106 | 11,630,010 | 11,682,638 | 11,595,970 | 11,583,106 |
| Midwest | 15,090,693 | 14,936,091 | 14,771,831 | 14,633,681 | 14,436,058 | 14,317,668 | 14,349,192 | 14,436,982 |
| South | 26,380,200 | 26,205,326 | 26,114,157 | 26,103,523 | 25,705,836 | 25,981,602 | 26,272,576 | 26,625,456 |
| West | 16,959,314 | 16,748,103 | 16,630,126 | 16,527,483 | 16,198,818 | 15,891,938 | 16,147,386 | 16,233,868 |

Data on overall population denominator was obtained from the U.S. Census Bureau’s estimates of the civilian U.S. population from January 1, 2016 to December 31, 2023. The 2016 to 2020 population denominator was estimated from the 2010 census. However, the 2021-2022 population denominator was estimated from the 2020 census. The 2016 to 2019 estimates may have underestimated the U.S. population compared with the estimates from the 2020 census. For example, the number of U.S. individuals aged 18-25 years as of July 2020 was estimated to be 35,137,978. But based on the 2020 census, the same number was estimated to be 35,575,999 (1.03 times higher). Thus, 2016-2019 denominators were multiplied by 1.03 to avoid the artificial abrupt jump in the population denominator between 2019 and 2020. The table above shows the calculated denominators used in the analysis.
